# International Medical Congress

**Published:** 1887-07

**Authors:** J. Taft


### International Medical Congress.

EXPLANATORY STATEMENT.

At the recent meeting of the American Medical Association at Chicago the following resolution was adopted by an almost unanimous vote :

" *Resolved*, That the regular graduates of such Dental and Oral Schools and Colleges as require of their students a standard of preliminary or general education, and a term of professional study equal to the best classes of medical colleges of this country, and embrace in their curriculum all the fundamental branches of medicine, differing chiefly by substituting practical and clinical instruction in Dental and Oral Medicine and Surgery instead of Clinical Medicine and Surgery be recognized as members of the regular profession of medicine, and eligible to membership in this Association on the same conditions and subject to the same regulations as other members."

This resolution is plain and comprehensive. Learning, however, that there is still uncertainty in the minds of some, as to the rights, privileges, and status of regular graduates of the class of Dental Colleges specified in the resolution, in the ninth International Medical College, it is deemed right and proper to say that the graduates of all Dental Colleges whose requirements conform to the above resolution are considered as members of the medical profession and are eligible to membership in the American Medical Association, and may register and take out a card of membership in the Congress according to general regulations, precisely upon the same terms and under the same conditions as those who hold the degree of M.D.

Dental practitioners, who according to the above resolution are not graduates, but are recommended by members of the Council, become members of the Congress upon invitation, authorized by the Executive Committee.

The members of the Seventeenth Section differ in no respect from those of any other Section of the Congress and have the same rights, and privileges, and status.

Without doubt a large proportion of those who have received notice that they will be invited are fully qualified according to the above resolution. Whether all are cannot now be determined, nor is it important that it should be. It is desirable to have present all who have been selected for membership; it is also hoped that those who are qualified according to the resolution will become members, and those who have not this special qualification, but have high attainments in the Science and Practice of Dentistry may become members as any other scientific men.

Invitations will soon be sent to all who have signified a willingness to accept an invitation, and though for many it is not necessary, yet it is thought best to adhere to the original plan.

Should there be any who have delayed complying with the request of the circular of Dr. F. H. Rehwinkel in regard to invitations, they are requested to comply speedily.

J. TAFT, of Section 17, I.M.C.

